# Pharmacists’ Perspectives on Their University Education: A Comparative Study of Graduates from Northern Sweden

**DOI:** 10.3390/pharmacy14070152

**Published:** 2026-10-17

**Authors:** Sofia Mattsson, Helena Norberg, Maria Gustafsson

**Affiliations:** Department of Medical and Translational Biology, Umeå University, SE-901 87 Umeå, Sweden; sofia.mattsson@umu.se (S.M.); helena.norberg@umu.se (H.N.)

**Keywords:** pharmacy, education, educational satisfaction, professional identity

## Abstract

This study evaluated educational satisfaction among pharmacists who graduated from Umeå University’s online pharmacy programs between 2019 and 2023, including the potential impact of the COVID-19 pandemic. Satisfaction levels were compared with those reported by graduates from 2015–2018, as well as between educational and job satisfaction. Data were collected through alumni surveys. Overall educational satisfaction among the pharmacy graduates was high. However, satisfaction levels were significantly lower than in the 2015–2018 cohort, with a smaller proportion reporting being very satisfied with the education (82.1% vs. 96.0%, *p* = 0.011). No statistically significant differences in educational satisfaction were observed between graduation groups defined in relation to the COVID-19 pandemic. Most respondents reported that the knowledge and skills acquired during the education were useful in their current job (77.4%), that they felt well prepared for professional practice (74.4%), that they would recommend the program to a prospective student (82.9%), and that the education provided them with a clear professional identity (80.8%). No significant differences between cohorts were observed for these outcomes. Higher educational satisfaction was associated with greater job satisfaction. Assessing educational satisfaction may provide valuable insights for the ongoing development of pharmacy education and support graduates’ preparedness for professional practice.

## 1. Introduction

Online education offers flexibility, enabling students to combine their studies with family and work commitments that might otherwise be difficult to accommodate [1,2]. Consequently, satisfaction with online education has become an important construct in higher education research. In this study, educational satisfaction refers to graduates’ overall subjective evaluation of their university education, including the extent to which the education meets their expectations and perceived needs. Evidence indicates that clear expectations, well-structured learning management systems (LMSs) and high-quality instructional materials are critical for successful online courses and educational satisfaction [3,4,5]. Withdrawal from online higher education is influenced by multiple factors, including course quality, academic preparedness and motivation, learning system characteristics, institutional support and satisfaction with the educational experience [6]. Educational satisfaction is associated with the perceived relevance of studies to future employment, employability, the student–staff ratio, teaching quality, and overall wellbeing [7,8,9]. Wellbeing is in turn influenced by the learning environment, the perceived meaningfulness of educational activities, and workload [10]. Educational satisfaction also contributes to the development of professional identity [11].

Against this background, the COVID-19 pandemic substantially altered the context of online education. The World Health Organization (WHO) declared COVID-19 a public health emergency of international concern on 30 January 2020 and characterised it as a pandemic on 11 March 2020 [12]. In response, countries worldwide introduced measures such as social distancing policies and school closures. By 12 March 2020, 46 countries had announced school closures, including 26 with nationwide closures [13]. Higher education was consequently disrupted worldwide, requiring universities to shift rapidly to remote teaching despite limited preparation and considerable pedagogical and organisational challenges [14]. 

Although Sweden did not impose a complete national lockdown, higher education was substantially affected. At Umeå University, where online teaching was already well established in the pharmacy programs, all on-campus activities were suspended. Laboratory sessions, oral communication and presentation exercises were cancelled or adapted, while traditional written examinations were largely replaced by take-home examinations. Pharmacy placements continued as usual, and on-campus activities were resumed after the pandemic. These challenges highlight the importance of examining whether the pandemic affected graduates’ educational satisfaction. 

Umeå University has extensive experience in delivering online pharmacy education, having admitted its first students in 2003. The program combines digital course delivery via an LMS with periodic on-campus sessions each semester. Prior to the COVID-19 pandemic, a survey was conducted in March 2019 among pharmacy graduates from Umeå University, Sweden, who had completed their studies between 2015 and 2018 [15]. These findings indicated a high level of educational satisfaction, which appeared stable over time when compared with an equivalent survey of graduates from 2006 to 2014 [15]. Despite these findings, longitudinal investigations of educational satisfaction in established online pharmacy educations remain scarce. Given the established importance of student satisfaction in online education, it is therefore pertinent to examine how satisfaction with online pharmacy education evolves over time.

Building on this earlier work, the present study aimed to evaluate educational satisfaction among pharmacists who graduated from Umeå University’s online pharmacy programs in Sweden between 2019 and 2023. In addition, the study sought to examine the potential impact of the COVID-19 pandemic on educational satisfaction within this cohort. Educational satisfaction was also compared over time with that of a previous cohort of graduates from 2015 to 2018. Finally, association between educational satisfaction and job satisfaction was explored.

## 2. Materials and Methods

### 2.1. Setting

At Umeå university, three pharmacy programs are offered: a three-year Bachelor of Science in Pharmacy program, a five-year Master of Science in Pharmacy program, and a two-year Master of Science in Pharmaceutical Science program. Graduates who hold a Bachelor of Science in Pharmacy degree are eligible to apply for the two-year master’s program to obtain a master’s degree. Five public universities in Sweden provide pharmacy education, and Umeå University is one of two delivering the education through an online format.

In Sweden, pharmacy education leads to two different professional degree levels: prescriptionists (with a bachelor’s degree), and pharmacists (with a master’s degree). Licences to practise as prescriptionist or pharmacist are issued by the National Board of Health and Welfare, and in 2024 there were 4588 registered prescriptionists and 3941 registered pharmacists in practice in Sweden [16]. In the remainder of this article, the term pharmacist will be used to refer to both professional categories.

The pharmacy curricula comprise courses in areas such as chemistry, biomedical sciences, pharmacology, pharmacotherapy, pharmaceutics, social pharmacy, clinical pharmacy, and pharmacy practice. The master courses entail more in-depth study that builds on the courses at the bachelor level. Each year, approximately 400 students graduate from pharmacy programs at Swedish universities, of whom around 75 are graduates from Umeå University [17].

### 2.2. Alumni Survey

A cross-sectional survey was conducted among graduates from the pharmacy programs at Umeå University, Sweden. All individuals who completed one of the three pharmacy programs between 1 January 2019 and 30 September 2023 were invited to participate. In total, 330 graduates were eligible during the study period. In October 2023, a paper-based questionnaire was distributed to the graduates. Current postal addresses were obtained from the university’s administrative records, which are regularly updated with information supplied by the Swedish Tax Agency, the authority responsible for citizens’ registration in Sweden. Participants could choose to return the completed questionnaire by mail using a prepaid envelope or to respond digitally by scanning a QR code. Two reminder e-mails were sent to the e-mail addresses registered in the university’s administrative system. Data collection took place during October and November 2023. Overall, 125 graduates responded to the questionnaire, either by post (*n* = 21) or digitally (*n* = 104), resulting in a response rate of 38%.

Previous alumni surveys have been conducted among pharmacy graduates at Umeå University, including one study of graduates from 2006–2014 [18] and another covering graduates from 2015–2018 [15]. In an earlier study, education satisfaction outcomes were compared between graduates from 2006–2014 and 2015–2018 [15]. In the present study, education satisfaction results were compared with those from the most recent alumni survey, i.e., graduates from 2015–2018 [15]. As the methodology applied in the current study was identical to that used previously, direct comparisons were possible.

The survey included questions regarding the graduates’ educational experiences, as well as their current employment and job satisfaction.

### 2.3. Data Analysis

Descriptive statistics were applied to summarise the dataset. For reference, characteristics of graduates included in the previous survey (graduation years 2015–2018) are also presented.

The following items—“I am very satisfied with the education,” “The knowledge and skills acquired during the education are useful in my current job,” “I would recommend the program to a prospective student,” “I felt well prepared to work as a pharmacist after completing the education,” and “The education gave me a clear professional identity”—were assessed using a five-point Likert scale (1 = strongly disagree, 2 = disagree, 3 = neither agree nor disagree, 4 = agree, 5 = strongly agree). For the purposes of analysis, responses were dichotomized into “disagree” (Likert scale points 1–3) and “agree/strongly agree” (Likert scale points 4–5).

Further, the item “My duties at work reflect the program” had dichotomous response options (yes/no). The question “If I were to choose today, I would start the same education at the same university” was dichotomized into “yes” (yes, the same education at the same university) and “no” (yes, the same education but at a different university; no, another education at the same university; no, another education at another university; or no, I would not start any education).

Graduates reporting satisfaction with their job were compared with those reporting dissatisfaction in relation to the questions: “I am very satisfied with the education,” “I felt well prepared to work as a pharmacist after completing the education,” and “The education gave me a clear professional identity”. These analyses were performed using chi-square tests.

To assess the potential impact of the COVID-19 pandemic on the survey outcomes, a subgroup analysis was conducted. The cohort was stratified into two groups based on year of graduation: those who graduated before or during the early phase of the pandemic (2019–2020) and those who graduated during the later phase and after the pandemic (2021–2023). Comparisons between the subgroups were conducted using chi-square tests.

Additionally, comparisons between the 2015–2018 and 2019–2023 graduation cohorts were conducted. Separate logistic regression models were fitted for each survey question, with the respective question included as the dependent variable. Year of graduation, current workplace, and country of birth were entered as categorical independent variables, while age and time since graduation were included as continuous independent variables.

Results are reported as odds ratios (ORs) with corresponding 95% confidence intervals (CIs). Statistical significance was set at *p* < 0.05. All analyses were performed using SPSS for Windows, versions 30 and 32.

## 3. Results

### 3.1. Participants’ Characteristics

Participant characteristics are summarized in Table 1. Respondents were predominantly female, with a mean age of 34 years. Around two thirds of the participating graduates were born in Sweden. Most respondents (67.5%) were employed in community pharmacies. A difference in workplace was observed between graduates with bachelor’s and master’s degrees, with the latter more frequently employed within county council services and the pharmaceutical industry. For reference, characteristics of graduates included in the previous survey (graduation years 2015–2018) are also presented in Table 1.

### 3.2. Educational Satisfaction 

When combining the responses “agree” and “strongly agree”, a majority (82.1%) of the graduates expressed a high level of overall satisfaction with their education. Table 2 summarises the respondents’ perceptions of their educational experience. A substantial proportion (77.4%) reported that the knowledge and skills acquired during their education were beneficial in their current employment, and 82.9% would recommend the program to prospective students. Furthermore, 74.4% indicated that they felt well prepared for professional practice as pharmacists upon graduation, while an even larger proportion (80.8%) reported that the program contributed to a strong sense of professional identity. Details regarding bachelor’s and master’s degree data are provided in Supplementary File Tables S1 and S2.

To assess whether the COVID-19 pandemic influenced the survey outcomes, a subgroup analysis was undertaken. The cohort was divided into two groups according to year of graduation: those who graduated before or during the early phase of the pandemic (2019–2020) and those who graduated during the later phase and after the pandemic (2021–2023), but where most of the studies were conducted during the pandemic. Table 3 presents the results of the subgroup analysis for the same items as reported above. No statistically significant differences were observed between the two groups for any of the items (*p* > 0.05). Similarly, no statistically significant differences were observed when the same analyses were performed separately for bachelor’s (Table S3) and master’s degree (Table S4) holders. 

When examining associations between educational satisfaction and job satisfaction, a significantly larger proportion of graduates who were satisfied with their education also reported being satisfied with their job (*p* = 0.003), compared with those who were not satisfied with their job. Further, there was a significantly higher percentage of graduates who felt well prepared to work as pharmacists (*p* = 0.015) and who thought that the education gave them a clear professional identity (*p* = 0.008) among those who reported being satisfied with their job. This contrasts with those who were not satisfied with their job. 

### 3.3. Comparison with a Previous Survey 

The results concerning educational satisfaction were compared with data from graduates completing their studies between 2015 and 2018, collected through a previous alumni survey [15]. The data is presented in Table 4. Across both graduation cohorts (2015–2018 and 2019–2023), most respondents expressed positive views regarding the relevance and value of their pharmacy education. Most graduates in both cohorts agreed that the knowledge and skills gained during their studies were useful in their current professional roles, that they would recommend the program to a prospective student, that their work duties aligned with their educational background, that they felt well prepared to work as pharmacists, that the education gave them a clear professional identity, and that they would start the same education if they were to choose again. 

The regression analysis revealed one statistically significant finding; graduates from 2015–2018 were more satisfied with the education (96.0% vs. 82.1%, *p* = 0.011), compared with those graduating in 2019–2023. For all other items, no statistically significant differences were identified between the two graduation periods.

## 4. Discussion

The results of the study showed that the overall educational satisfaction among pharmacy graduates from Umeå University in Sweden was high. Educational satisfaction appears not to have been affected by the COVID-19 pandemic. Although a relatively high level of educational satisfaction was reported, educational satisfaction has significantly decreased since the last investigation at Umeå University (graduation years 2015–2018). The same questions regarding educational satisfaction were used in both investigations, making comparisons possible. In addition, no substantial changes have been made to the pharmacy programs during the time period, except for changes applied during the COVID-19 pandemic, as described below. However, course content, assignments and examinations are subject to regular review and revision.

Higher levels of educational satisfaction were reported in the present study compared with studies from, for example, Saudi Arabia [19], Jordan [20] and Australia [21]. One aspect of educational satisfaction could be the willingness to choose pharmacy again. Most of the respondents in the present study indicated that they would choose pharmacy education again at the same university if given the opportunity, suggesting satisfaction with both the program and the discipline. This finding is consistent with previous research on educational satisfaction among pharmacy students. For example, a study conducted in Lebanon reported that 66% of respondents would select pharmacy again [22], while a study from the United States found that most participants agreed or strongly agreed that they would choose pharmacy as their major again [23]. However, direct comparisons across studies should be made with caution due to differences in study contexts and survey methodologies.

The analysis of the potential impact of the COVID-19 pandemic did not detect a statistically significant difference in educational satisfaction between the two graduation groups. However, the results should be interpreted with caution, as they may be influenced by the limited sample size and challenges in defining a precise cut-off for pandemic exposure. The pandemic had a substantial effect on society including higher education and affected students, academic staff and educational institutions [14]. Universities were required to make a rapid transition to remote teaching, often under significant time constraints and with limited opportunities for preparation. Since the pharmacy programs at Umeå University were already delivered online, the impact was less pronounced than in campus-based education. However, previously on-campus activities, such as laboratory experiments, role-play and oral presentations, were affected and had to be either cancelled or adapted to an online format, substantially decreasing opportunities for practical work and face-to-face social interactions. This may have negatively impacted on the students’ practical, social and communicative skills, but this has not been formally assessed. The on-campus activities were resumed after the pandemic. 

Several studies have examined students’ experiences of online learning during the COVID-19 pandemic. While online learning was perceived as an effective instructional approach, it was considered less effective than face-to-face teaching for developing clinical skills and social competences [24]. Studies also identified the quality of online learning during the pandemic, including opportunities for active engagement, feedback, and skill development, as a key determinant of student satisfaction [24,25,26].

Compared with graduates from the earlier cohort (graduation years 2015–2018), the latter reported higher levels of satisfaction with the education compared with those from the present cohort (graduation years 2019–2023) (Table 4). The results showed a clear deviation from previous trends, where educational satisfaction among pharmacy graduates at Umeå University remained stable between 2006 and 2018 but has since declined, albeit from a relatively high level [15].

Most respondents in the present study perceived that the education provided them with a clear professional identity and the regression analysis showed no statistically significant difference compared with the previous cohort (graduation years 2015–2018) (*p* = 0.324) (Table 4). The development of a professional identity has been identified as a potential challenge in distance education programs [27]. The cultivation of professional identity is a central objective of vocational education, and the education needs to support individual professional identity development [28]. Development of professional identity is a complex process and is influenced by several educational strategies, including curriculum design, reflective learning activities, and interprofessional education [29,30,31,32,33,34,35]. Previous research has highlighted the importance of experiential learning through internships, as well as the integration of theoretical and practical knowledge, in supporting students’ professional identity formation [29]. To facilitate this process, Umeå University has incorporated reflective professional development portfolios into the pharmacy curriculum [31]. In addition, interprofessional learning activities involving pharmacy students and students from other healthcare professions have been established within the medical faculty at the university. Such initiatives have been shown to play an important role in fostering professional identity development [35]. A noteworthy deviation is that a lower proportion of the respondents in the 2015–2018 cohort answered the question about professional identity and this may influence the comparison (Table 4). Although the reason for the missing responses is unclear, it may reflect uncertainty regarding the concept of professional identity, whereas the aforementioned initiatives may have increased understanding of the concept, and consequently, the response rate to this question in the 2019–2023 cohort. Previous research has indicated that pharmacy students may face challenges in establishing a professional identity, emphasizing the need for educational strategies that actively support professional identity formation within pharmacy programs [36]. Ongoing changes in pharmacists’ roles and responsibilities may shape how professional identity is developed and perceived. Therefore, pharmacy education has an important role in supporting students’ adaptation to these changes.

A majority of the respondents indicated that the knowledge and skills acquired during the education were useful in their current job, that they would recommend the program to a prospective student, that they felt well prepared for professional practice as pharmacists after graduation, and that the duties at work reflected the education. Regarding these aspects, there were no significant differences between the two cohorts when taking into account potential confounders in the regression analysis (Table 4). The results suggest that the educational content is relevant and effectively prepares students for their professional roles. The perceived relevance of studies to future employment has been reported as an indicator for educational as well as job satisfaction [7]. A recent study among pharmacy graduates in South Korea found that those who perceived a higher alignment between their education and current professional roles reported higher levels of career satisfaction [37].

Job satisfaction among the same respondents (graduation years 2019–2023) was evaluated in a previous study [38]. Although job satisfaction was relatively high, the results showed a decrease in job satisfaction compared to the cohort with graduation years 2015–2018 [38]. The results in the present study showed that there was an association between educational satisfaction and job satisfaction, i.e., a significantly larger proportion of graduates who were satisfied with their education (*p* = 0.003), who felt well prepared to work as pharmacists (*p* = 0.015) and who thought that the education gave them a clear professional identity (*p* = 0.008) also reported being satisfied with their job, compared with those who were not satisfied with their job. The reported decrease in job satisfaction parallels the reported decrease in educational satisfaction. However, given the cross-sectional methodology of the present study, no conclusions regarding causality can be drawn. The decrease in job satisfaction may reflect increasing strain within the healthcare system, placing greater pressure on pharmacists to safeguard patient safety [39]. The observed decline may indicate a heightened awareness of professional challenges, such as high workload, stress, and limited opportunities for career advancement [38]. Furthermore, dissatisfaction with professional practice may extend to the perceptions of educational experiences.

This study has several strengths and limitations. A major strength is the ability to examine educational satisfaction over an extended period, including the potential impact of the COVID-19 pandemic. However, the study is limited by the difficulty in defining a precise cut-off for pandemic exposure. The period 2019–2020 was used as an approximation, and it was assumed that students graduating in 2020 were less substantially affected by the pandemic, given that they were in their final phase of their education (March to June 2020). However, graduation year serves only as an indirect proxy for exposure to pandemic-related educational changes and it does not fully capture the extent or duration of exposure to pandemic-related restrictions. As a result, the analysis cannot provide robust evidence regarding the impact of COVID-19 on educational satisfaction, and the findings need to be interpreted with caution.

The use of the same education satisfaction questions across different cohorts made it possible to compare results over time. Nevertheless, education satisfaction is a subjective concept that may be interpreted differently depending on personal preferences and cultural background, which can affect individuals’ perceptions of educational satisfaction. In addition, recall bias may have influenced the results. Due to limitations in the methodology, factors affecting educational satisfaction, such as student–staff ratio, teaching quality, class sizes, and course funding, were not explored in the present study and therefore the underlying reasons for the observed decrease in educational satisfaction could not be analysed further. Respondents for whom employment was not the primary occupation (those reporting to be students or on parental leave) were also included in the job satisfaction analyses, as they could provide responses based on part-time employment or previous professional experience as pharmacists.

Moreover, the findings may not be entirely generalisable to all pharmacists in Sweden, as the study population included graduates from only one university. The comparatively low response rate (38%) may limit both the representativeness of the sample and the generalisability of the findings. Selection bias cannot be excluded, as individuals with a more favourable view of their pharmacy education may have been more willing to participate in the survey than those with less positive experiences. Taken together, the low response rate represents a potential limitation that may affect comparisons between the cohorts. 

The analyses were exploratory and aimed to identify potential associations across educational outcomes. Therefore, the findings should be interpreted with caution, particularly considering the increased risk of Type I error associated with multiple comparisons. Potential confounders were incorporated in the regression analysis, but possible residual confounding cannot be excluded. Also, combining “neither agree nor disagree” with “disagree” in the performed regression analysis may result in loss of information and potentially misclassify respondents with neutral opinions.

Examining students’ satisfaction with their education may provide educators with valuable insights for refining the pharmacy curriculum and enhancing students’ preparedness for professional practice. In addition, students’ satisfaction with their studies has been identified as an important factor influencing learning outcomes [40], underscoring the importance for educators to investigate educational satisfaction among students and graduates. Student satisfaction is also considered as a key factor associated with dropout in online higher education [6] and understanding educational satisfaction can thus help educators work with student retention.

## 5. Conclusions

Overall, respondents remained satisfied with their education, even though the satisfaction levels were significantly lower compared with previous investigations among pharmacy graduates from Umeå University in Sweden. Regarding the COVID-19 pandemic, no statistically significant differences in educational satisfaction were detected between the defined graduation groups in relation to the pandemic.

Most graduates in both cohorts (graduation years 2019–2023 and 2015–2018) agreed that the knowledge and skills gained during their studies were useful in their current professional roles, that they would recommend the program to a prospective student, that their work duties aligned with their educational background, that they felt well prepared to work as pharmacists, that the education gave them a clear professional identity, and that they would start the same education if they were to choose again. There were no statistically significant differences between the cohorts (graduation years 2015–2018 and 2019–2023) regarding these items. 

Individuals reporting higher levels of job satisfaction also tended to express greater satisfaction with their education. However, the cross-sectional methodology of the present study does not support casual interpretations of the association between satisfaction in the two domains.

## Figures and Tables

**Table 1:** Characteristics of participating pharmacy graduates (graduation year 2019–2023), with data from the 2015–2018 cohort included for comparison (data from [15]).

Characteristics	All Graduates (Graduation Year 2019–2023) *n* = 125	Bachelor’s Degree (Graduation Year 2019–2023) *n* = 69	Master’s Degree (Graduation Year 2019–2023) *n* = 56	All graduates (Graduation Year 2015–2018) *n* = 94
Gender (%)				
Women	87.9	85.6	90.9	92.6
Age (years)				
Mean (standard deviation)	34.0 (8.8)	33.3 (9.0)	35.0 (8.6)	35.0 (8.6)
Range	21–58	21–57	24–58	
Country of birth (%)				
Sweden	65.0	68.7	59.3	77.2
Outside Sweden	35.0	31.3	40.7	22.8
Country of living (%)				
Sweden	99.2	100.0	98.1	98.9
Outside Sweden	0.8	0.0	1.9	1.1
Time since graduation (years)				
Mean (standard deviation)	1.8 (1.4)	1.7 (1.5)	1.9 (1.5)	2.7 (1.1)
Occupation (%)				
Employed	80.0	78.3	87.5	74.5
Student	12.0	15.9	3.6	8.5
Parental leave	5.6	2.9	8.9	17.0
Other *	2.4	2.9	0.0	0.0
*Workplace (%)*				
Community pharmacy	67.5	82.6	48.2	76.3
Hospital pharmacy	3.3	5.8	1.8	4.3
County council **	9.8	4.3	16.1	6.5
Pharmaceutical company	9.8	0.0	21.4	3.2
Other ***	9.6	7.3	12.5	9.7

* Other includes unemployed and sick leave. ** County councils are responsible for the public healthcare systems in Sweden. *** Other includes drug product manufacturing, dose dispensing pharmacy, university, and municipality.

**Table 2:** Questions regarding educational satisfaction among pharmacy graduates. Number and proportion of graduates choosing the different options in the survey are presented.

Question	Number and Percentage of Respondents *n* (%)				
	Strongly Disagree	Disagree	Neither Agree nor Disagree	Agree	Strongly Agree
I am very satisfied with the education (*n* = 123)	1 (0.8)	9 (7.3)	12 (9.8)	60 (48.8)	41 (33.3)
The knowledge and skills gained during education are beneficial in my current job (*n* = 124)	3 (2.4)	14 (11.3)	11 (8.9)	63 (50.8)	33 (26.6)
I would recommend the program to a prospective student (*n* = 123)	5 (4.1)	8 (6.5)	8 (6.5)	31 (25.2)	71 (57.7)
I felt well prepared to work as a pharmacist after completing the education (*n* = 125)	3 (2.4)	13 (10.4)	16 (12.8)	55 (44.0)	38 (30.4)
The education gave me a clear professional identity (*n* = 125)	3 (2.4)	7 (5.6)	14 (11.0)	43 (34.4)	58 (46.4)

**Table 3:** Questions regarding education satisfaction among pharmacy graduates in the two subgroups (graduation year 2019–2020 and 2021–2023). Results presented show the number and proportion of students answering agree/strongly agree to the questions.

Question	Graduation Year 2019–2020 *n* (%)	Graduation Year 2021–2023 *n* (%)	*p*-Value
I am very satisfied with the education (*n* = 123)	34/42 (82.0)	66/80 (82.5)	0.833
The knowledge and skills gained during education are beneficial in my current job (*n* = 124)	32/42 (76.2)	63/81 (77.8)	0.842
I would recommend the program to a prospective student (*n* = 123)	34/41 (82.9)	67/81 (82.7)	0.977
I felt well prepared to work as a pharmacist after completing the education (*n* = 125)	32/43 (74.4)	60/81 (74.1)	0.967
The education gave me a clear professional identity (*n* = 125)	36/43 (83.7)	64/81 (79.0)	0.528

**Table 4:** Logistic regression including different questions regarding education satisfaction. Graduation year 2015–2018 and 2019–2023. Results presented show the number and proportion of students answering agree/strongly agree *, or yes ** to the questions.

Question	Graduation Year 2015–2018 *n* (%)	Graduation Year 2019–2023 *n* (%)	Odds Ratio ^1^	95% Confidence Interval	*p*-Value
I am very satisfied with the education *	89/93 (96.0)	101/123 (82.1)	0.216	0.066–0.704	0.011
The knowledge and skills acquired during the education are useful in my current job *	76/92 (82.6)	96/124 (77.4)	0.935	0.432–2.025	0.865
I would recommend the program to a prospective student *	86/93 (92.5)	102/123 (82.9)	0.553	0.204–1.503	0.245
I felt well prepared to work as a pharmacist after completing the education *	75/92 (81.5)	93/125 (74.4)	0.829	0.393–1.748	0.623
The education gave me a clear professional identity *	59/64 (92.2)	101/125 (80.8)	0.558	0.174–1.782	0.324
The duties at work reflect the education **	67/93 (72.0)	93/124 (75.0)	0.659	0.332–1.306	0.232
If I were to choose today, I would start the same education at the same university **	72/92 (78.3)	83/123 (67.5)	0.622	0.308–1.258	0.187

^1^ Controlled for categorical variables; current workplace (categories community pharmacy (ref) and other ^2^); country of birth (categories Sweden (ref) and outside Sweden); and the continuous categories age and time since graduation (continuous category). ^2^ Other includes hospital pharmacy, county council, pharmaceutical company, drug product manufacturing, dose dispensing pharmacy, university, and municipality.

## Data Availability

The original contributions presented in this study are included in the article. Further inquiries can be directed to the corresponding author.

## Supplementary Materials

The following supporting information can be downloaded at: https://www.mdpi.com/article/10.3390/pharmacy14070152/s1. Table S1: Questions regarding educational satisfaction among pharmacy graduates (bachelor); Table S2: Questions regarding educational satisfaction among pharmacy graduates (master); Table S3: Questions regarding education satisfaction among pharmacy graduates (bachelor) in the two groups (graduation year 2019–2020 and 2021–2023); Table S4: Questions regarding education satisfaction among pharmacy graduates (master) in the two groups (graduation year 2019–2020 and 2021–2023).
