# Supplementary material for: Pharmacists’ Perspectives on Their University Education: A Comparative Study of Graduates from Northern Sweden
Source: Pharmacy (Basel). 2026 Oct 17;14(7):152. doi: 10.3390/pharmacy14070152 (PMC13708153; doi:10.3390/pharmacy14070152)
Supplement: Supplementary file 1 [file pharmacy-14-00152-s001.zip › pharmacy-4573696-supplementary.pdf]

Supplementary Materials

Details regarding bachelor's and master's degree data

Pharmacists' Perspectives on Their University Education: A Comparative Study of Graduates from Northern Sweden

Sofia Mattsson, Helena Norberg and Maria Gustafsson

Table S1. Questions regarding educational satisfaction among pharmacy graduates (bachelor). Number and proportion of graduates choosing the different options in the survey are presented.

	Strongly disagree	Disagree	Neither agree or disagree	Agree	Strongly agree
I am very satisfied with the education (n=68)	1 (1.5)	4 (5.9)	10 (14.7)	32 (47.1)	21 (30.9)
The knowledge and skills acquired during the education are useful in my current job (n=68)	2 (2.9)	4 (5.9)	8 (11.8)	38 (55.9)	16 (23.5)
I would recommend the program to a prospective student (n=68)	4 (5.9)	4 (5.9)	7 (10.3)	15 (22.1)	38 (55.9)
I felt well prepared to work as a pharmacist after completing the education (n=69)	2 (2.9)	6 (8.7)	14 (20.3)	34 (49.3)	13 (18.8)
The education gave me a clear professional identity (n=69)	2 (2.9)	4 (5.8)	10 (14.5)	26 (37.7)	27 (39.1)

Table S2. Questions regarding educational satisfaction among pharmacy graduates (master). Number and proportion of graduates choosing the different options in the survey are presented.

	Strongly disagree	Disagree	Neither agree or disagree	Agree	Strongly agree
I am very satisfied with the education (n=55)	0 (0.0)	5 (9.1)	2 (3.6)	28 (50.9)	20 (36.4)
The knowledge and skills acquired during the education are useful in my current job (n=56)	1 (1.8)	10 (17.9)	3 (5.4)	25 (44.6)	17 (30.4)
I would recommend the program to a prospective student (n=55)	1 (1.8)	4 (7.3)	1 (1.8)	16 (29.1)	33 (60.0)
I felt well prepared to work as a pharmacist after completing the education (n=56)	1 (1.8)	7 (12.5)	2 (3.6)	21 (37.5)	25 (44.6)
The education gave me a clear professional identity (n=56)	1 (1.8)	3 (5.4)	4 (7.1)	17 (30.4)	31 (55.4)

Table S3. Questions regarding education satisfaction among pharmacy graduates (bachelor) in the two groups (graduation year 2019-2020 and 2021-2023). Results presented show the number and proportion of students answering agree/strongly agree to the questions.

	Graduation year 2019-2020 n (%)	Graduation year 2021-2023 n (%)	p-value
I am very satisfied with the education	17/23 (73.9)	36/45 (80.0)	0.567
The knowledge and skills gained during education are beneficial in my current job	17/22 (77.3)	37/46 (80.4)	0.763
I would recommend the program to a prospective student	17/22 (77.3)	36/46 (78.3)	0.927
I felt well prepared to work as a pharmacist after completing the education	16/23 (69.6)	31/46 (67.4)	0.855
The education gave me a clear professional identity	17/23 (73.9)	36/46 (78.3)	0.687

Table S4. Questions regarding education satisfaction among pharmacy graduates (master) in the two groups (graduation year 2019-2020 and 2021-2023). Results presented show the number and proportion of students answering agree/strongly agree to the questions.

	Graduation year 2019-2020 n (%)	Graduation year 2021-2023 n (%)	p-value
I am very satisfied with the education	17/19 (89.50)	30/35 (85.7)	0.694
The knowledge and skills gained during education are beneficial in my current job	15/20 (75.0)	26/35 (74.3)	0.953
I would recommend the program to a prospective student	17/19 (89.5)	31/35 (88.6)	0.920
I felt well prepared to work as a pharmacist after completing the education	16/20 (80.0)	29/35 (82.9)	0.792
The education gave me a clear professional identity	19/20 (95.0)	28/35 (80.0)	0.129
